# Supplementary material for: A Non-Conventional Platinum Drug against a Non-Small Cell Lung Cancer Line
Source: Molecules. 2023 Feb 10;28(4):1698. doi: 10.3390/molecules28041698 (PMC9964417; doi:10.3390/molecules28041698)
Supplement: Supplementary file 1 [file molecules-28-01698-s001.zip › molecules-2195296-SI.pdf]

A non-Conventional Platinum Drug Against a non-Small Cell Lung Cancer Line

Jéssica D. Silva [1], Joana Marques [1], Inês P. Santos [1], Ana L.M. Batista de Carvalho [1], Clara B. Martins [1], Raquel C. Laginha [1], Luís A.E. Batista de Carvalho [1*], M. Paula M. Marques [1,2]

[1] Molecular Physical-Chemistry R&D Unit, Department of Chemistry, University of Coimbra, 3004-535 Coimbra, Portugal

[2] Department of Life Sciences, Faculty of Science and Technology, University of Coimbra, 3000-456 Coimbra, Portugal

* Correspondence: labc@ci.uc.pt; Tel.: +351 239 854 462

Supplementary Material

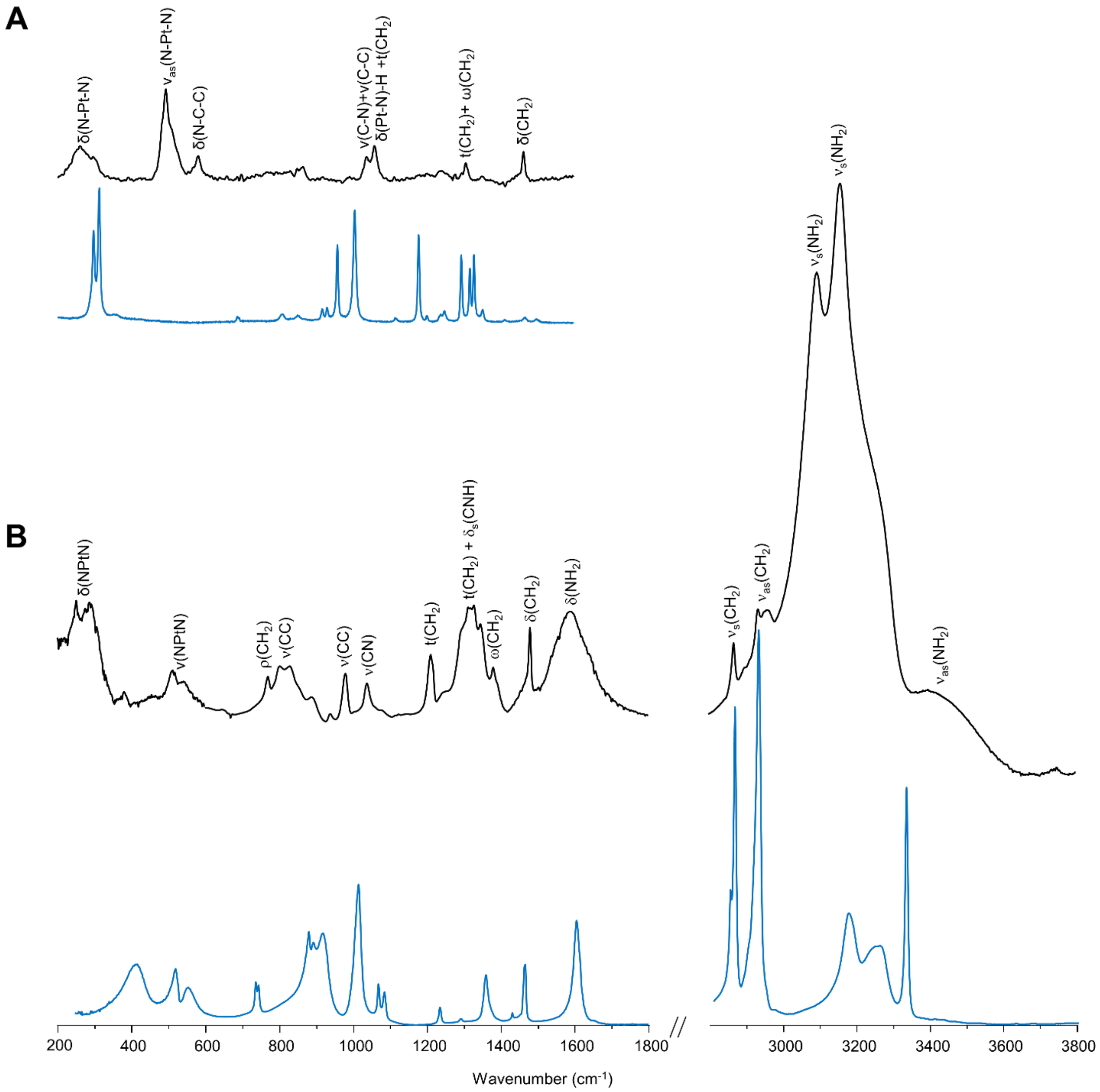

Figure S1. Raman (A) and FTIR (B, far- and mid-infrared regions) spectra of $[Pt_2Put_2(NH_3)_4]^{4+}$ (black line) and putrescine (blue line) (solid state).

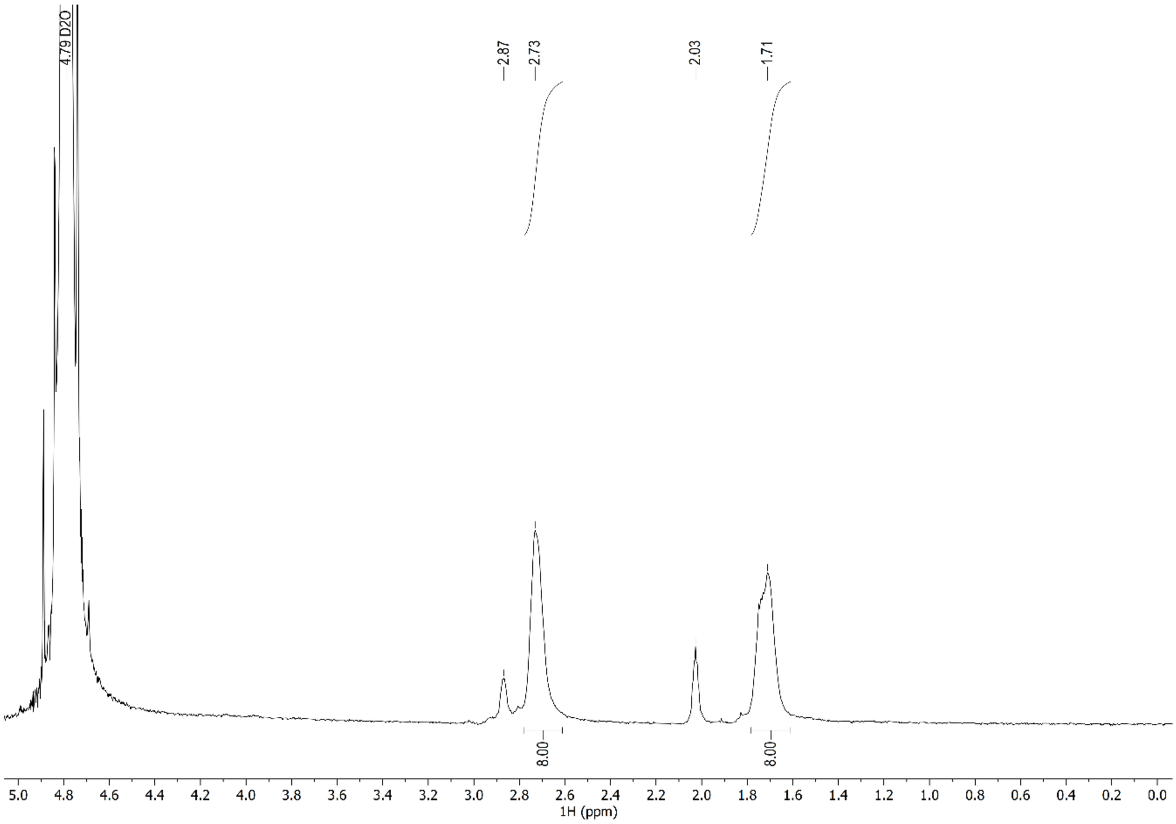

Figure S2. NMR spectrum of $[Pt_2Put_2(NH_3)_4]^{4+}$ in a D_2O solution.
